# Human Genetic Variation Influences Enteric Fever Progression

**DOI:** 10.3390/cells10020345

**Published:** 2021-02-06

**Authors:** Pei Yee Ma, Jing En Tan, Edd Wyn Hee, Dylan Wang Xi Yong, Yi Shuan Heng, Wei Xiang Low, Xun Hui Wu, Christy Cletus, Dinesh Kumar Chellappan, Kyan Aung, Chean Yeah Yong, Yun Khoon Liew

**Affiliations:** 1 School of Postgraduate Studies, International Medical University, Bukit Jalil, Kuala Lumpur 57000, Malaysia; Ma.PeiYee@student.imu.edu.my; 2 School of Pharmacy, International Medical University, Kuala Lumpur 57000, Malaysia; TAN.JINGEN@studentimuedu.onmicrosoft.com (J.E.T.); Hee.EddWyn@studentimuedu.onmicrosoft.com (E.W.H.); DYLAN.YONGWANG@studentimuedu.onmicrosoft.com (D.W.X.Y.); Heng.YiShuan@studentimuedu.onmicrosoft.com (Y.S.H.); LOW.WEIXIANG@studentimuedu.onmicrosoft.com (W.X.L.); xunhui.wu@outlook.com (X.H.W.); CHRISTY.CLETUS@studentimuedu.onmicrosoft.com (C.C.); 3 Department of Life Sciences, International Medical University, Kuala Lumpur 57000, Malaysia; Dinesh_Kumar@imu.edu.my; 4 Department of Pathology, International Medical University, Kuala Lumpur 57000, Malaysia; ukaung2548@gmail.com; 5 Department of Microbiology, Faculty of Biotechnology and Biomolecular Sciences, Universiti Putra Malaysia, Selangor 43400, Malaysia; yongcheanyeah@hotmail.com

**Keywords:** enteric fever, *Salmonella* typhoidal species, human genetic variants

## Abstract

In the 21st century, enteric fever is still causing a significant number of mortalities, especially in high-risk regions of the world. Genetic studies involving the genome and transcriptome have revealed a broad set of candidate genetic polymorphisms associated with susceptibility to and the severity of enteric fever. This review attempted to explain and discuss the past and the most recent findings on human genetic variants affecting the progression of *Salmonella* typhoidal species infection, particularly toll-like receptor (TLR) 4, TLR5, interleukin (IL-) 4, natural resistance-associated macrophage protein 1 (NRAMP1), VAC14, PARK2/PACRG, cystic fibrosis transmembrane conductance regulator (CFTR), major-histocompatibility-complex (MHC) class II and class III. These polymorphisms on disease susceptibility or progression in patients could be related to multiple mechanisms in eliminating both intracellular and extracellular *Salmonella* typhoidal species. Here, we also highlighted the limitations in the studies reported, which led to inconclusive results in association studies. Nevertheless, the knowledge obtained through this review may shed some light on the development of risk prediction tools, novel therapies as well as strategies towards developing a personalised typhoid vaccine.

## 1. Introduction

Enteric fever is caused by the Gram-negative bacillus, *Salmonella* typhoidal species namely *S. enterica* serotype Typhi which is responsible for typhoid fever and *S. enterica* serotype Paratyphi which results in paratyphoid fever, and is considered as a major worldwide health problem [1,2,3]. The disease is characterised by prolonged fever, generalised fatigue, headache and anorexia. In general, the outbreak of enteric fever mainly results from faecal–oral transmission through the ingestion of food and water contaminated with human excreta [4,5]. Hence, the risk factors associated with enteric fever could be related to poverty and social inequality, e.g., through lack of sanitation and hygiene, human demographics and behaviour, as reported elsewhere [6,7].

The occurrence of typhoid fever extends throughout the globe. It is prevalent primarily in developing countries with poor sanitary conditions. Typhoid fever is native to Africa, the Caribbean, Asia, Oceania and Latin America. However, most of the cases originate from Laos, Vietnam, China, Pakistan, Bangladesh, Nepal, India and Indonesia [8]. Within these nations, typhoid fever is mostly reported in underdeveloped regions. According to the model-based estimation carried out by Antillón et al., in 2015, approximately 17.8 million of typhoid fever cases per year are estimated in people who are living in low and middle-income countries [6].

In addition, the ‘global burden of disease 2016′ project shows that there is a slight decrease in the global age-standardised typhoid fever death rate, which dropped by 21.1% from 2.155 deaths per 100,000 in 2006 to 1.7 deaths per 100,000 in 2016. Furthermore, paratyphoid fever deaths decreased by 6.6% from 137.3 thousand deaths to 128.2 thousand deaths in 2016 [9]. Although enteric fever cases declined in a number of countries in recent years, there were still high incidences of typhoid fever in some regions in Africa, as estimated by the adjusted incidence rate, which indicated that children in the age range of 2–14 years have the greatest typhoid fever burden [10]. Importantly, since the year 2000, its increasing resistance to ciprofloxacin treatment has also attracted considerable attention.

**Figure 1:**
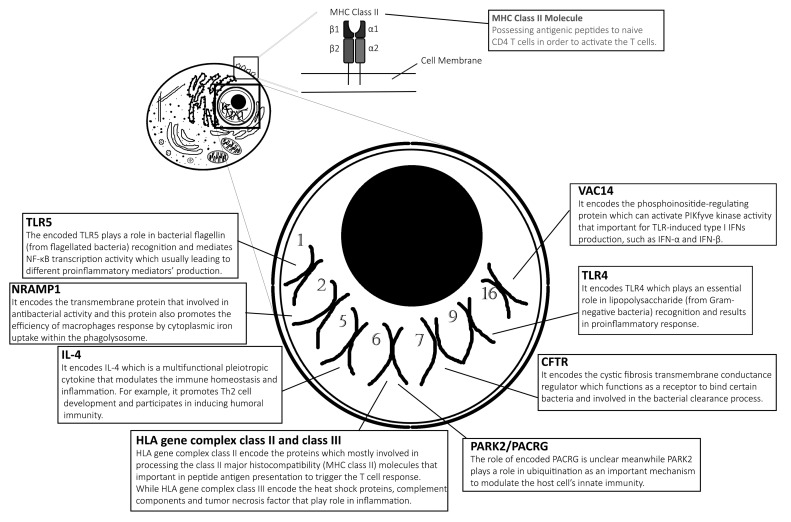
Genes responsible for diverse biological functions. Alteration of the specific genes might affect the susceptibility or severity to *Salmonella* typhoidal species infection as described and discussed in the content of this review. Notes: This illustration of the eukaryotic cell does not reflect its actual features, while it is just purely for illustration and only seven pairs of chromosomes are represented in this diagram.

Typhoid vaccines are developed to protect individuals from enteric fever. One of them is an injectable polysaccharide (PS) vaccine (also known as ViCPS vaccine), an inactivated subunit vaccine composed of long chains of sugar molecules that make up the surface capsule of *S.* Typhi. Another vaccine is an edible vaccine which was also developed from live attenuated mutant strains of *S.* Typhi Ty21a. However, not all vaccine recipients receive adequate protection and benefits from the immunisation against this intracellular *Salmonella* spp. It is believed that the genetic variation of the recipients plays a major role in modulating the antibody response to typhoid vaccine [11]. They found that alteration of genes involved in PS recognition, signalling ligands and receptors are the main genetic polymorphisms associated with typhoid vaccination outcomes. In addition, genetic variation also influences the individual’s susceptibility to enteric fever. For example, *PARK2/PACRG* polymorphism is found to be among the candidate gene variants that are greatly associated with enteric fever [12]. In this review, we summarise the most recent and some past findings on human genetic variants affecting biological functions in influencing the outcome of *Salmonella* typhoidal species’ infection. Table 1 lists the influence of human genetic factors in the development of enteric fever and the role of the genes are illustrated in Figure 1.

## 2. Host Genetic Variants

### 2.1. Toll-Like Receptor 4: Initiates the First Line of Defense

It is well known that Toll-like receptors (TLRs) are used by many innate immune cells to recognise the microbes upon binding to its pathogen-associated molecular patterns (PAMPs), such as components of microbial membranes, cell walls, proteins and oligonucleotides. This interaction complex may lead to the activation of innate immune cells in promoting the production of pro-inflammatory mediators and inducing an appropriate expression of receptor molecules, such as costimulatory molecules, cytokine or chemokine receptors and integrin molecules. Altogether, the activation of TLRs in innate immunity is also one of the crucial steps to initiate the subsequent adaptive immune response which has been elaborately reviewed by others [25,26,27,28,29,30,31]. In short, the activated innate immune cells, particularly dendritic cells (DCs) secrete an array of cytokines which include the interleukin-6 (IL-6), that allows effector T cells to subdue the suppressive effect of regulatory T cells. Collectively, these mechanisms mediate the activation of T helper (T_H_)-cell immunity and induces the maturation of B-cells which are responsible for the clearance of bacteria [32,33,34,35]. In the case of TLR4 activation, upon lipopolysaccharide (LPS) binding, it bestows the early mounting of nonspecific immune response to infections. For *S.* Typhi, the TLR4 not only recognises the LPS molecules, but also the porin expressed on the outer membranes of the bacterial cell wall. Cervantes-Barragán et al. found that *S.* Typhi OmpC and OmpF porins were important to provide signalling via TLR4, to activate the innate immune cells and B cells in enhancing the *S.* Typhi porins-specific antibodies production [36]. The more detailed description about the immune response after the infection by *S*. Typhi or other species of *Salmonella* may be referred through reviews elsewhere [35,37,38,39].

Thus, it is not surprising that polymorphisms in *TLR4* might cause predisposition to susceptible infection by *S.* Typhi. Several other studies have reported that mutation in *TLR4* gene or absence of this gene increases the susceptibility of *S*. Thyphimurium in mice, which mimics human typhoid infection [13,31,40,41,42,43,44,45,46]. Poltorak et al. and Hue et al. identified that the *TLR4* gene mutation causes defective TLR4 signalling [29,44], which might result in a lack of inflammatory cytokine production and decrease in the shaping of an adaptive immune response [47]. *TLR4* Asp299Gly is a missense mutation that causes the substitution of the aspartic acid residue with glycine at amino acid 299 in the fourth exon of *TLR4*, whereas *TLR4* Thr399Ile is a mutation that occurs by the substitution of a non-conserved threonine residue with isoleucine at amino acid 399 [30,31,48]. Polymorphisms at these amino acids alter the PAMPs-binding site of the extracellular domain of the TLR4 receptor and enhance the susceptibility to enteric fever [13,30,31]. Arbour et al. found that the individual with both *TLR4* Asp299Gly and *TLR4* Thr399Ile polymorphisms would be hyporesponsive when challenged with LPS and was less efficient in eliciting distinct innate immune responses [31]. The polymorphic variant of *TLR4* also results in an increased susceptibility to other microbial infections, such as *Toxoplasma gondii*, Cytomegalovirus, *Neisseria meningitides*, *Mycobacterium leprae* and other intracellular pathogens [14,49,50]. However, further discussion about this *TLR4* polymorphism association with non-*Salmonella* infectious diseases is not within the scope of this review. We suspect that the polymorphisms in *TLR4* might result in intracellular killing mechanisms to be affected. To the extent of our knowledge, intracellular TLR4 signalling is able to increase the release of nitric oxide within the phagocytes [45,51], and hence mutant *TLR4* alleles may result as ineffective in bactericidal activity on intracellular *S.* Typhi by nitric oxide.

In a study performed on a Malay population in Malaysia, the significantly higher frequency of the mutant G (299 Gly) allele and mutant allele T (399 Ile) in susceptible individuals, as compared to that in the healthy controls, showed an association between these mutant alleles with typhoid susceptibility. The genotype frequency of *TLR4* Thr399Ile heterozygous variant was approximately two-fold higher than in healthy controls. On the other hand, the mutant G allele at the Asp299 locus was approximately three times higher in the typhoid susceptible group than in the control subjects [13]. Bhuvanendran et al., reported that the prevalence of *TLR4* Asp299Gly and Thr399Ile polymorphisms was 8.9% and 7.2%, respectively, in the typhoid susceptible population, which compares to 1.8% and 3.6% in the corresponding normal population. The co-segregation of these alleles was observed in 2% of normal controls and 3.6% in typhoid susceptible individuals. However, another study demonstrated that monocytes from Asp299Gly heterozygotes did not influence the ability of monocytes in LPS recognition, where the activation of TLR4 downstream signalling still occurs [52]. This finding is in contrast with the data obtained from different research groups as discussed above. For example, Arbour and colleagues reported that airway epithelial cells obtained from individuals heterozygous for the *TLR4* mutations did not respond to LPS stimulation [31]. This highlights the complexity of the TLR4 system in different cells and tissues. The detailed mechanisms and evidence on different immunological aspects of the TLR4 system at distinct cell types are poorly understood. Furthermore, differences in immune responses have not been evaluated with other TLR4 ligands, such as purified porin.

Other than in a Malay population, there were 13 TLR4 polymorphisms reported in a study done among Caucasian and Dutch populations [53,54]. TLR4 *Asp299Gly* mutations are common variants with a frequency of >10% in the Caucasian population and as high as 21.5% in Ghanaian Africans [55,56]. *TLR4* Asp299Gly and *TLR4* Thr399Ile polymorphisms commonly co-segregate as well as inherit together in European whites but not in African population [57,58,59]. The lack of co-segregation for Asp299Gly and Thr399Ile alleles in the African population could be one of the reasons for the higher incidence and mortality rates of enteric fever. This is because a study had indicated that the presence of the *TLR4* Asp299Gly allele alone is associated with an increased severity of Gram-negative bacterial infection [46].

Despite the common functional *TLR4* mutants (Asp299Gly and Thr399Ile) found in Malaysia, Europe and Africa, it came as a complete surprise that both these alleles were reported to be absent or present at a low frequency in the Vietnamese population, especially *TLR4* Asp299Gly [31,44]. In contrast, DNA sequencing studies performed on a Vietnamese population observed that the exonic polymorphisms actually related to the mutations in the N-terminal leucine-rich repeat (LRR) region within extracellular domain of TLR4, when compared to reference sequences (NCBI accession number AF 177765) [44]. These mutations in the LRR region, particularly *TLR4* Ser73Arg, showed significant association with enteric fever because it could disturb the phosphorylation of TLR4, thus altering the downstream signalling of an inflammatory mediator activation. However, the question of whether the *TLR4* Ser73Arg could be a main SPN effect on typhoid susceptibility if this study repeated in large sample size is still not known yet.

Collectively, the data obtained from molecular genetic studies indicated that the presence of rare missense mutations in the *TLR4* gene, particularly those that influence the extracellular domain of TLR4, may affect the immune response to enteric fever; and the polymorphism may also be different according to the varying ethnic backgrounds. However, it is currently unknown whether there is any influence on the new functional *TLR4* mutants on the host response to *S*. Typhi infection. To date, there have not been many studies on TLR4 polymorphisms in a larger geographic range for the *S*. Typhi infected patients that would give rise to clinical diversity. Other than TLR4, TLR2 polymorphism and its relationship with typhoid fever has also been studied. Only one such study led by Sivaji’s team at Tamil Nadu, India, in 2016, showed that the TLR2 polymorphism induced susceptibility to typhoid infection, since it was found in 10% (2/20) of typhoid patients. This TLR2 acts as a receptor that recognises LPS and may also recognise lipoproteins in order to trigger a strong immune response.

### 2.2. Toll-Like Receptor 5: Recognises Bacterial Flagellin

TLR5 is believed to have the ability to recognise the flagellin of *S.* Typhi and thereby stimulate an immune response similarly to in TLR4 activation, which had been evidenced via animal studies [26,33,60,61]. The availability of functional TLR5 is needed to play an important role against flagellated bacterial infection [62,63]. For example, various polymorphisms in the *TLR5* gene by introducing a premature stop codon (*TLR5^392STOP^*) within the PAMPs binding domain are correlated to different degrees of interleukin 6 (IL-6) production, which may affect innate and adaptive immune responses [64,65]. It has been reported that single nucleotide polymorphisms (SNPs) in *TLR5^392STOP^* are associated with Legionnaires’ disease caused by *Legionella pneumophila* [64]. However, this would not be the same story for enteric fever. In recent years, experiments have been carried out to investigate the relationship between the host susceptibility to enteric fever and the loss of TLR5 or its polymorphism *TLR5* on chromosome 1. Surprisingly, most of their findings showed discrepancies from the study focused on *TLR4* polymorphisms as discussed above. They showed that the variation in the *TLR5* gene did not affect individual susceptibility to typhoid fever [22,66]. For instance, Senthilkumar et al. reported that the *TLR5^392STOP^* polymorphism did not show any association with clinical manifestations in Indian patients with typhoid fever and asymptomatic typhoid carriers. Before that, Dunstan et al. had already analysed the stop codon polymorphism of TLR5 in patients with enteric fever compared to healthy Vietnamese individuals (565 patients; 281 controls) for the samples collected between 1995 and 2002 [22,23,66]. Similarly, this study also did not find any significant difference between both studied groups. Therefore, it could be postulated that a mutation at the PAMPs domain binding site of TLR5 (individuals with the TLR5 stop codon) might have no correlation with susceptibility to enteric fever progression.

### 2.3. Natural Resistance-Associated Macrophage Protein 1: Kills Intracellular Pathogens

Natural resistance-associated macrophage protein 1 (NRAMP1) is a transmembrane protein found in the endosomes and lysosomes of monocytes and macrophages, which are encoded by the *NRAMP1* gene (also called as *SLC11A1*). This plays an important role in exhibiting its antimicrobial activity and is also involved in iron homeostasis for macrophages to enable macrophage functions properly [67]. Variations in *NRAMP1* gene at chromosome 2 is therefore expected to cause a broad range of susceptibility to infection [1,68,69]. Interestingly, a recent study by Cunrath et al. unravelled that NRAMP1 could decrease the growth of nontyphoidal *Salmonella* by limiting the magnesium availability for *Salmonella* as shown in their vivo assay [70]. Nevertheless, polymorphisms within the *NRAMP1* gene are not expressed in correlation with typhoid fever. According to Dunstan et al., there were no allelic associations that were identified among the *NRAMP1* alleles and typhoid fever susceptibility [24]. Therefore, none of the homozygotes and heterozygotes of the *NRAMP1* variants are at increased risk of typhoid fever. However, some studies showed that *NRAMP1* polymorphisms may be associated with infectious diseases such as tuberculosis (TB), leprosy, rheumatoid arthritis and Crohn’s disease [68,69,71,72]. According to Medapati et al., 3′-UTR, INT4, D543N and 5′-(GT)n polymorphisms of *NRAMP1* were significantly associated with TB among west Africans [73]. Another study conducted by Brochado et al. reported the 274C/T polymorphism in exon 3 and the 469 + 14G/C polymorphism in intron 4 of the *NRAMP1* gene which were found to be associated with susceptibility to leprosy [74].

### 2.4. VAC14: Acts as Core Subunit of Lipid Kinase in Signalling the Type I IFNs Production

VAC14 is a phosphoinositide-regulating protein which activates PIKfyve kinase activity by forming a regulatory complex with PIKfyve and the gene that encodes *VAC14* is located on chromosome 16 [75,76]. PIKfyve, a class III lipid kinase, is crucial in TLR-induced type I IFN production. Although the polymorphisms of *TLR5* and *NRAMP1* do not show impact on enteric fever susceptibility, *S*. Typhi clearance is currently reported to be influenced by *VAC14* variations [16]. One of the more serious SNPs of the *VAC14* gene is an allele of rs8060947, which is strongly associated with susceptibility to *S*. Typhi invasion and decreased *VAC14* expression in Vietnamese individuals [16]. There is another study which demonstrated that the variation in *VAC14* for Kenyan children could also be readily associated with the increased risk of bloodstream infection from *Escherichia coli*, *Acinetobacter* spp., nontyphoidal *Salmonella* as well as Gram-positive *Streptococcus pneumoniae* [77,78]. A genome-wide association study (GWAS) complemented with high-throughput human in vitro susceptibility testing (Hi-HOST) showed the mutations in the *VAC14* gene and it has been linked to an increase in plasma membrane cholesterol which in turn facilitates the *Salmonella* Typhi docked firmly to the host cell [16]. There is plenty of literature showing that plasma membrane cholesterol content is an essential factor for *E. coli*, *S. pneumoniae* and toxins of *Acinetobacter* species to enter the host cells [79,80,81]. Therefore, the enhanced expression of *VAC14* could be responsible for its protection against *S*. Typhi and suggests that rs8060947 could be a biomarker for enteric fever disease risk prediction.

As mentioned above, VAC14 is also one of the essential protein subunits for PIKfyve in signalling the type I IFNs production. Therefore, the impact of *VAC14* allele of the variant rs8060947 might interrupt the cells in producing type I IFNs which can either be beneficial or otherwise to the host defence system. However, polymorphisms of *VAC14* in relationship with type I IFNs production is yet to be investigated for the *S*. Typhi infection models [82]. In general, type I IFNs comprise IFN-α and IFN-β, in which are both important in antiviral response; but with abnormal upregulation, may lead to the progression of systemic lupus erythematosus (SLE). Until recently, IFN-β has been reported as an important cytokine that stimulates the prevention of the dissemination of *S*. Typhimurium and enhances *Salmonella* clearance after the host has been treated with polyinosine–polycytidylic (poly I:C) [83]. A notable feature of the IFN-β is that it also acts as an inducer by enhancing the IFN-γ production from CD4^+^ cells, especially the T_H_1 cells [38].

It is well known that IFN-γ plays a critical role in innate immunity as well as adaptive immunity [84,85]. Previously, IFN-γ was detected in high titer in patients with *S*. Typhi bacteremia as reported by Sheikh et al. [86]. Of note, Bhuiyan and colleagues also demonstrated the increased IFN-γ response after stimulation with different typhoid antigens and this was consistent with the findings of Sheikh et al. [38]. Other than that, the increased IFN-γ response was observed in a subject from Nepal who was infected with *S.* Typhi and *S.* Paratyphi A, while there was a report which showed that a child with a complete loss of IFN- γR1 was killed by *S.* Typhi infection [87,88]. The role of IFN-γ has been evaluated by Nairz, who showed that IFN-γ could reduce the iron uptake of macrophage through transferrin receptor [89]. Hence, in other words, it increased the efflux of iron; and at the same time, strengthened the ability of macrophages to kill intracellular pathogens including *Salmonella* [90]. The bacteria cannot grow well in the absence of iron. The IFN-γ also induces the expression of other proteins (such as NRAMP1 and lipocalin 2) that are involved in the reduction in cytoplasmatic iron or other divalent cations in the infected host cells [91,92]. Other roles of IFN-γ such as phagolysosomal maturation, oxidative and nitrosative burst, autophagy and decreasing the levels of tryptophan to reduce the growth of intracellular *Salmonella* can be observed in the literature [51,82,93,94,95,96].

### 2.5. Variable Number of Tandem Repeat (VNTR) in Interleukin 4: Generating Variation in Gene Expression

Variable number of tandem repeats (VTNRs), also known as single-copy mini-satellites, consists of consecutive occurrences of nucleotide sequences at a particular locus [97]. VNTRs sequences play a major role in regulating the transcription, translation and function of proteins when located within the coding area of certain genes. This, in turn, could lead to severe forms in different diseases [97]. The mode of how VTNRs induce changes in *IL-4* expression, which is located on chromosome 5, will be described in this section. Generally, VNTR polymorphisms in *IL-4* intron 3 often occur in four different ways; allele with two repeats (2R), three repeats (3R), four repeats (4R) and rarely with one repeat (1R) [15]. Therefore, these mutations seem to be associated with susceptibility to and/or progression towards enteric fever upon *S*. Typhi or *S*. Paratyphi infections [98]. In fact, there are not many studies on the association of VNTR *IL-4* polymorphisms with other infectious diseases. We only acknowledge that some VNTR *IL-4* polymorphisms could affect the host susceptibility to brucellosis which was caused by intracellular *Brucella melitensis* [99]. As far as we know, any VNTR polymorphism at *IL-4* intron 3 is also associated with other immunological diseases, such as rheumatoid arthritis, immune thrombocytopenic purpura, systemic lupus erythematosus, multiple sclerosis and knee osteoarthritis [98,100,101]. This is because IL-4 is one of the major cytokines responsible for both innate and adaptive immunity which influences various cell types. It has been demonstrated that the polymorphism of 3R2R in *IL-4* most likely results in the reduction in protection against *S*. Typhi and *S*. Paratyphi, conferring a higher risk in the development of enteric fever [15]. In contrast, the polymorphism of 3R3R, 3R1R, and 2R2R does not contribute to any susceptibility towards enteric fever [15]. It was suggested that the *IL-4* VNTRs polymorphism might affect the transcriptional activity, including the higher expression of *IL-4*, leading to a shift in the Th1/Th2 balance toward Th2 [15,101]. It is well known that the protective immune response against *S.* Typhi is the maturation of Th1 cells, apart from the B cells [38]. Immunisation studies with iron-regulated outer-membrane proteins of *S.* Typhi has also shown that the protection against *Salmonella* infection was initiated by the Th1 cells [102]. This is because the Th1 cells may drive macrophages to M1 polarisation [103,104]. It is well known that the activated M1 macrophages play a main role in killing the intracellular pathogen [105]. Through this mechanism, individuals with VNTR polymorphism in *IL-4*, especially, the 3R2R genotype, are more susceptible to *S*. Typhi or *S*. Paratyphi infection.

### 2.6. PARK2/PACRG: Ubiquitination

*PARK2/PACRG* genes are located on chromosome 6. The *PARK2* gene is responsible for encoding ‘parkin’, which plays a major role in ubiquitination [106]. The specific function of *PACRG* gene is still unclear. Polyubiquitination by parkin for the targeted protein is commonly needed to initiate the proteasome-mediated protein degradation pathway [107]. Both ubiquitination and degradation might play important roles in many biological functions, and the failure of this processes could ultimately lead to many human diseases which are not limited to only non-infectious diseases. Studies have found an association between this protein degradation pathway and the ability of *Salmonella* spp. to invade the host cells [108,109]. For example, upon contact with the host cells, Rho GTPases Cdc42 and Rac1 in the host cell are activated by *Salmonella* effectors encoded by *sopE* and *sptP* during the entry process. In general, SopE resulted in extensive membrane ruffling and the actin cytoskeletal network reorganisation of the host cell which is important for bacterial uptake into host cell, while SptP restores the host cell architecture. The SopE can be rapidly degraded while SptP is slowly degraded within the host cell via an ubiquitin/proteasome-mediated degradation pathway. Therefore, any mutation in *PARK2* that alters its ubiquitination ability will undoubtedly affect the half-life of SopE. Consequently, this inhibits cellular recovery after bacterial infection, and results in persistent membrane ruffling which allows the *S.* Typhi or *S.* Paratyphi to grow continually inside the host cells as it easily enters the cells [110,111].

As aforementioned, parkin encoded by *PARK2* was observed to play a role in ubiquitination, while most importantly, this mechanism also modulates the host cell’s innate immunity. Ubiquitination is an essential part in innate immunity by signalling the cells for the proper secretion of various signalling mediators [112]. Up to now, it is believed that parkin might act as a mediator for the production of two key pro-inflammatory cytokines: IL-6 and monocyte chemoattractant protein 1 (MCP-1). In this, IL-6 contributes to systemic inflammatory response or differentiate the activated B cells, and MCP-1 attracts monocytes into the infected area. This has been proven by the downregulation of the *PARK2* gene expression in Schwann cells, monocyte-derived macrophages and THP-1 macrophages [113]. Moreover, it has also been shown that polyubiquitination contributes to autophagic pathway by coating the intracellular pathogen with ubiquitin which enables it be readily targeted for autophagy. Details regarding the autophagy in the host cell’s innate immunity can be reviewed in other literatures [114,115,116]. Indeed, a few in vitro and in vivo studies have been performed to elucidate the functional mechanism of the *PARK2* gene in autophagic immunity against *M. tuberculosis*, *S.* Typhimurium and *Streptococcus pyogenes* [117,118,119]. The expression of PARK2 was observed to recruit more ubiquitin and ubiquitin-binding autophagy adaptors to the bacteria, which resulted in the reduction in bacterial survival, whereby increasing the infected cell survival. Therefore, it is not surprising that there might be an association between *PARK2* alleles and innate immune response. Notably, the polymorphisms of *PARK2/PACRG* genes were found to be associated with susceptibility to infectious diseases including typhoid and paratyphoid fever [12,106,120,121]. Additionally, different variants of *PARK2/PACRG* genes were also reported in association with susceptibility to infection caused by *M. leprae*, for example, SNP of rs1333955, rs10806768 and rs9355403.

In the last decade, a case-control study was conducted in the Jatinegara district of Jakarta, Indonesia. This study examined the polymorphisms in *PARK2/PACRG* genes at the shared 5′ regulatory region of *PARK2* and *PACRG.* This was performed to study both the environmental factors and genetic determinants towards the susceptibility to enteric fever. Four SNPs (*PARK2_*e01(−2599), *PARK2*_e01(−697), *rs1333955* and *rs1040079*) were used for this study in evaluating the polymorphic variants of the *PARK2/PACRG* genes. This was carried out in 116 enteric fever patients, with 337 as fever controls and 332 as community controls. The findings showed genetic variations at this targeted gene region, which might be correlated with the individual susceptibility to enteric fever. By combining the results of Hardy–Weinberg equilibrium of each SNPs and association study, only one SNP (allele T of *PARK2*_e01(−2599)) among the 4 SNPs analysed was found to be significantly—but weakly—associated with enteric fever (OR: 1·51; CI: 1·02–2·23, *p* = 0.03). Thus, the mutation in *PARK2/PACRG* genes, especially the variant *PARK2*_e01(−2599) has been suggested as one of the risk factors in the development of enteric fever and leading to the prolonged half-life of *Salmonella* effectors protein [12].

### 2.7. Cystic Fibrosis Transmembrane Conductance Regulator: Secretion Channel and Pili Receptor

The *CFTR* gene found on human chromosome 7 encodes the cystic fibrosis transmembrane conductance regulator (up to 1480 amino acids) which functions as a protein channel throughout various types of glandular cells for the secretion of enzymes, sweat, mucus, saliva and tears [122]. Mutations in this gene can result in a lethal genetic disorder known as cystic fibrosis [123,124,125].

Interestingly, CFTR protein also functions as a receptor for type IVB pili of *Salmonella* Typhi in order to adhere to the intestinal mucosa cells which facilitates the bacterial translocation process into the intestinal submucosa [126]. Afterward, *S.* Typhi might induce more intestinal epithelial cells to express its CFTR protein and this could be an essential step for the development of typhoid fever [127]. An in vitro study using isogenic cells has demonstrated that the mutant with phenylalanine deleted at residue 508 (∆508 or F508del mutation) is less susceptible to *S.* Typhi infection as compared to cells expressing wild-type *CFTR* [126]. Therefore, F508del mutation is believed to render a selective advantage against *S.* Typhi or *S.* Paratyphi infection to the host [17]. Another study using an animal model has demonstrated that the homozygous F508del mice are immune to the *S.* Typhi translocation into their gastrointestinal submucosa, thereby protected from enteric fever [126]. However, the distribution of F508del mutation in the human populations is uneven throughout the world, and it is uncommon to observe the F508del genotypes in Asian populations. The other form of *CFTR* mutation could be found to provide a selective advantage against *S.* Typhi infections.

In years past, *CFTR* intragenic polymorphic microsatellites analysis indicated that IVS8CA alleles 181 (CA_16_) and 183 (CA_17_) on intron 8 of the *CFTR* gene were significantly associated with resistance to enteric fever and van de Vosse et al. also identified that both IVS8CA genotypes of 181/181 and 181/183 have a protective effect for typhoid fever in a case-control study in Indonesia [17]. In an attempt to identify the contribution of functional mutated CFTR protein to protection against enteric fever, the same research team conducted a study in the Jatinegara district of east Jakarta (Indonesia), by analysing the blood samples positive for enteric fever and blood specimens from healthy control subjects [18]. As expected, the sequence variations in the *CFTR* gene of the Indonesian population were identified and the distribution of alleles and genotypes of typical polymorphisms were detected between the control subjects and enteric fever patients. However, interestingly, these DNA sequence polymorphisms showed no correlation between the IVS8CA repeat genotypes and susceptibility to enteric fever. In contrast, they revealed that IVS8 TG repeat genotypes are associated with protection against enteric fever, especially individuals who possess one of the *CFTR* variations or more than one *CFTR* variations (such as TG_n_ repeat genotype TG_11_TG_12_, 2562T > G genotype TG, Q1352H mutation, the alleles of IVS8 TG_13_ or TG_15_ and the allele of IVS8 T_5_) might be more resistant to *S.* Typhi or *S.* Paratyphi than other groups in the population [18].

### 2.8. MHC Class II and Class III: Antigen Presentation and Other Innate Immunity Responses

Over the last decade, the major-histocompatibility-complex (MHC) polymorphism has been recognised as a factor determining the various infectious disease susceptibility. MHC molecules are the glycoproteins on the cell surface of eukaryotic cells that play a crucial role in the immune system, autoimmunity and reproductive success [128,129]. The genes that encode this MHC molecule are located within the human leukocyte antigen (*HLA*) gene complex region either as class I or class II at chromosome 6. Another gene complex that can be found between them is *HLA* class III which consists of genes encoding tumour necrosis factor (TNF)-α, lymphotoxin (LT)-α and other gene products which do not play the same role as MHC glycoproteins [130]. Variation in *HLA* regions might result in a different degree of resistance or susceptibility to infectious diseases as discussed elsewhere [131,132,133,134]. In 2001, the polymorphism at *HLA* class II and class III regions have been extensively investigated to correlate with typhoid fever in the Vietnamese population [19]. According to Dunstan et al., the presences of both TNF*1 (−308) and HLA-DRB1*04 alleles in an individual might result in protection from typhoid fever. After a few years, Dunstan et al., also reconfirmed that variants of the *HLA-DRB1* gene are a main key for individual resistance to typhoid fever [20]. The application of imputation-based fine-mapping across the extended *HLA* region provided a confident datum to show that HLA-DRB1*04:05 allele as a protective factor against typhoid fever with its genotyped single nucleotide polymorphism (SNP), rs7765379. However, the alleles of HLA-DRB1*0301/6/8, HLA-DQB1*0201-3 and TNFA*2 (−308) have been reported to be associated with increased susceptibility to typhoid fever [132,133]. On the other hand, a study had demonstrated that individuals with the allele of HLA-DRB1*12021 were found to be protected from the severe outcomes of enteric fever [135]. The severity of infection was also affected by the mutation at the region of HLA class III, especially the *TNFA* gene. According to Elahi et al., guanine is substituted by adenosine in the synthesis of TNFA*2 (−308) in TNF-*α* polymorphism. Thus, the production of TNF-*α* is stimulated or markedly increased directly due to the presence of TNFA*2 (−308) allele. Consequently, some unwanted immune responses, which include shock, elevated temperature and sleepiness, could be induced by bacterial endotoxin, interleukin-1 and interleukin-6 when the titre of circulating TNF-α is extremely high for the individual with TNFA*2 (−308) polymorphism [136]. Therefore, the TNFA*2 (−308) allele is mostly thought to be associated with the severity of enteric fever instead of susceptibility. Furthermore, in contrast, a study in Vietnam, and a case-control study from Indonesia conducted by Ali and colleagues clearly indicated that there was no association of susceptibility to enteric fever and TNFA polymorphisms, particularly *TNFA (−308)* [21]. We could speculate that the severity of *Salmonella* typhoidal species infection might vary significantly between individuals based on *TNFA* and *HLA-DRB1* polymorphisms.

## 3. Limitations

### 3.1. Samples Size and Population

In this review, many gene polymorphisms have indicated a significant association among the susceptibility to, resistance to and the severity of enteric fever. However, the source of information for this review was mostly from the genome-wide profiles of genetic and gene expression studies having small sample sizes, which precludes the inclusion of multiple populations. This could lead to false-positive or false-negative results that might have hidden true associations due to a lack of statistical power. Foremost, the presence of low-frequency and rare alleles in small populations may miss inferences of genetic association for enteric fever. In other words, some DNA polymorphisms associated with enteric fever could exist but are unlikely to be statistically significant when the study is small in size. Thus, the large sample size should be included in order to confirm the level of significance. However, a large sample size does not always hold in practice for certain phenomena, whereby low enteric fever incidences are found in some countries and certain specimens are not readily obtainable. With a lack of diversity in the human population included in the association study and having insufficient reference sequences for the diverse population could also result in the incorrect imputation of genetic polymorphisms in correlation with enteric fever susceptibility or severity. It is well known that allelic variation in gene association with infectious diseases progression could be differently demonstrated in multiple populations. For example, different ethnicities will have unique polymorphisms conferring the development of infectious diseases as observed in malaria and tuberculosis. In addition, the environmental factor also influences the population genetic background. As a result, the individuals within the same geographic region from the same ancestry are less genetically different from one another compared to those who originated from a different geographic region. Hence, some false results regarding polymorphism association with enteric fever might occur due to this mixture of ethnicity and environmental factors. Therefore, precautionary steps should be taken during the data interpretation. In addition, some of the genetic variants correlating with susceptibility or resistance to *S.* Typhi infection as mentioned in this review have not been independently replicated in large studies across different populations.

### 3.2. Other Biological Specimens

Another important variable in the studies that causes a drawback is the limited types of biological samples obtainable, and the lack of an accompanying database of causative *S.* Typhi or *S.* Paratyphi variants when studying the human genetic polymorphisms in association with enteric fever. Most of the previous studies have used blood samples as these are an easily accessible biological sample, whereas a few of them utilised cell lines such as cervical cancer cells and intestinal mucosal cells for the investigation of gene mutations associated with *Salmonella* typhoidal species infection. In the expression quantitative trait loci (eQTL) analysis, it was indicated that gene expression variances at certain alleles have been shown over distinct tissues and cell types as reflected in the context of infection. Therefore, the statistical method is an indispensable need to unravel the signature genes expression from a rare cell subtype if the whole blood sample is used in the study. There are a few tools which can be applied to address this issue, for example the ARIEL test, cohort allelic sums test, sequence kernel association test, as well as FUN-LDA algorithm along with an R package. However, there still is a risk where the gene polymorphisms of the small proportion of cell subtype would not be detected upon the analysis of the whole blood sample. Furthermore, the *Salmonella* typhoidal species infection can also interact with microfold cells, liver macrophages (Kupffer cells), as well as other mononuclear phagocytic cells in lymphoid follicles and spleen, in addition to cells in the whole blood or peripheral blood samples. We should acknowledge that diverse cell types would behave differently even though they have an identical genome. The epigenetic polymorphisms provide an example of this phenomenon, where epigenetic modification through DNA methylation or histone methylation, ubiquitination acetylation or phosphorylation might be found in the host cells in response to *Salmonella* typhoidal species infection. In addition, the loci with alleles that differ at a single base have been discovered for macrophage and dendritic cells in several studies. Because of that, any cells which interact with *Salmonella* typhoidal species could be one of the factors in determining susceptibility to enteric fever for each individual. On the other hand, the variants of *S*. Typhi were reported elsewhere to possess variation in its virulence. The severity of the *S*. Typhi invasive infection might be implied by the strength of the virulence. It is a fact that the genetic susceptibility or resistance to infectious disease is also determined by pathogen genotypes. In this way, depending on the analysis of diverse biological specimens and the *Salmonella* typhoidal genotypes involved, more information about the genetic and allelic association in enteric fever could be revealed. However, some of the specimens are not easily accessible.

### 3.3. Other Approaches

**Figure 2:**
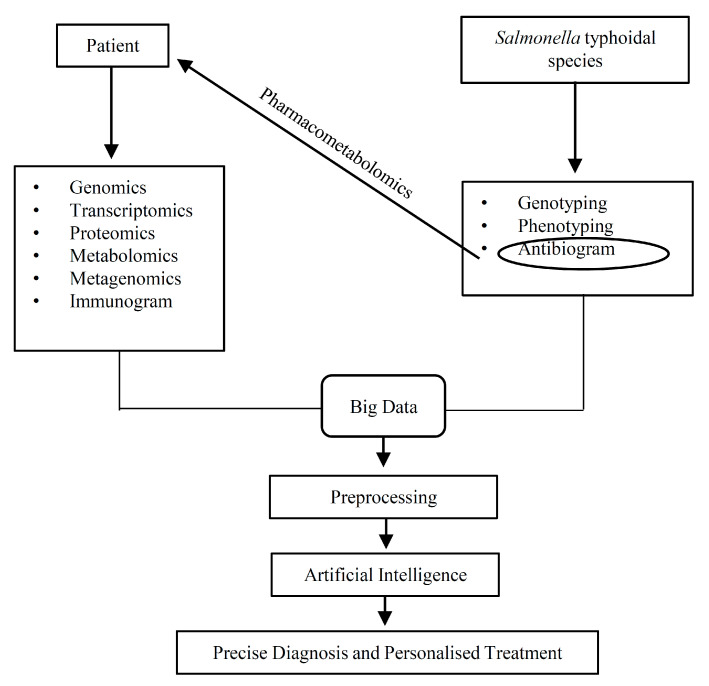
Integration of multiple approaches for biomarkers that are used as a precise diagnostic tool and also to formulate treatment plans.

Several gene polymorphisms reported here are less involved in the integration of other -omics technologies. There were only a few GWASs which have been integrated with eQTL analysis to identify the promising gene polymorphism that may underlie susceptibility to *Salmonella* typhoidal species infection. The rationale for integrating the -omics approaches is to complement each other, so that the specific gene polymorphisms associated with the susceptibility to or severity of enteric fever could be confidently pinpointed and provide fundamental biological information under the holistic picture. As aforementioned, epigenetic modification could be found in the genome of the host cells, and the non-coding RNA molecules are also involved in regulating the gene expression for host cell immune response upon infection with pathogen. Therefore, the current approach must integrate with epigenomic techniques such as TET-associated bisulfite sequencing (TAB-seq), oxidative bisulfite sequencing (oxBS-seq), nanopore sequencing, and chromatin immunoprecipitation followed by sequencing (ChIP–seq) to produce the epigenomic maps of the DNA methylation and modified histone landscapes. The current studies are also still lacking in the single-cell RNA sequencing (scRNA-seq) analysis of their samples. The power of this approach is that it offers an ability to investigate the mRNAs as well as non-coding RNA molecules in single cells. As described above, different cells will behave differently, probably due to its unique transcriptome, and thus we foresee the application of scRNA-seq in understanding the single-cell SNPs correlated with enteric fever populations. In addition, molecular typing and antibiogram profiling for *S.* Typhi and *S.* Paratyphi could be included in the study. The data might then be useful, particularly, as personalised and precision medicine have garnered much attention lately. Furthermore, this will allow us to have accurate diagnosis and personalised treatment. As illustrated in Figure 2, the integration of the characteristics of *Salmonella* and ‘-omics’ datasets along with immunoprofiling from respective patients have allowed the exploitation of available biological information into useful and informative outcomes after the pre-processed data are fed into a machine learning model. Such knowledge may reduce the risk of a severe outcome stratification of individuals exposed to *Salmonella* typhoidal species and allows to more precisely pinpoint the management and needs of individual groups of patients, thereby reducing enteric fever-related complications. As a key example of *VAC14* allele of the variant rs8060947 patient, antibiotics such as ciprofloxacin might be prescribed for the clearance of non-multidrug resistant *Salmonella* sp. together with IFN-γ.

## 4. Conclusions and Perspectives

Collectively, this review provided an appraisal of the contributory role of each polymorphism in the susceptibility to and severity of enteric fever. In addition, the role of some aforementioned genes has then been further discussed in the context of biological function which were interconnected with other biomolecular components. Gene polymorphisms associated with the severity of *Salmonella* typhoidal species infection implies its influence on immune response to typhoid vaccination. Therefore, the knowledge of allele variants (such as *TNF-α* polymorphisms) which lead to unwanted immune response after a challenge with salmonella, may help to predict typhoid vaccine effectiveness and facilitate the development of individual vaccination strategies. For example, the identification of TNFA*2 (-308) polymorphism as a biomarker could be used to predict individuals who might be at risk for typhoid vaccine-related adverse reactions. On the other hand, the polymorphisms of VAC14 or TLR4 as markers may help decide if there is the need to include immunostimulatory cytokines as adjuvants, especially IFN-γ and IL-6 during the vaccination. Combined together, these might increase the effectiveness of typhoid vaccines for those patients with the polymorphisms of VAC14 or TLR4. However, the comprehensive view of the enteric fever progression remains to be elucidated. This should include the integration of different -omics data such as vaccinomics, proteomics and metabolomics other than genetic data and transcriptome. Due to the availability of cloud computing, large -omics data could be simultaneously analysed and large-scale collaboration could be established across different fields. There also is a need to include relevant cell types for the -omics data analysis as well. The genetic and virulence background of the causative *S.* Typhi or *S.* Paratyphi for infected patients must be considered when studying the human genetic polymorphisms association with infection. This would be great benefit for interpreting the association between human gene variants with the susceptibility or severity of enteric fever. Nonetheless, there might be a lot of challenges during multiple data analysis, interpretation and validation. Despite these challenges, the integrative analysis will lead to the better understanding of the severity of enteric fever from the perspectives of human genetic polymorphisms or bacterial variants. The further studies may also provide insights into human genetic susceptibility to *Salmonella* typhoidal species infection which could be translated into new tools for risk prediction of *S.* Typhi or *S.* Paratyphi infection and might have an impact on the development of novel therapeutic drugs.

## Figures and Tables

**Table 1:** The influence of gene variants on enteric fever.

Gene ^a^	Location of Gene	Influence of Variants	Reference
**Gene Variants that Affect the Enteric Fever Outcomes**			
***TLR4***	Chromosome 9	Missense mutation in *TLR4* (threonine → isoleucine substitution at position 399 of the amino acid sequence) or (aspartate → glycine substitution at position 299 of the amino acid sequence) is evidenced to be associated with an increasing risk for *Salmonella* infection and severity of enteric fever.	[13,14]
***IL-4***	Chromosome 5	Variable number of tandem repeat polymorphisms of 3R2R at *IL-4* could be a genetic predisposition factor for *S.* Typhi or *S.* Paratyphi infection.	[15]
***VAC14***	Chromosome 16	The polymorphism rs8060947 in *VAC14* gene renders it susceptible to infection.	[16]
***PARK2/PACRG***	Chromosome 6	Mutation in *PARK2* results in a single-nucleotide polymorphism of *PARK2*_e01(−2599) which shows the weak association and susceptibility to typhoid fever and paratyphoid fever.	[12]
***CFTR***	Chromosome 7	Polymorphic dinucleotide repeats in the intron or exon of the *CFTR* gene and also in the single nucleotide variant, whereas, polymorphisms poly-T at *CFTR* gene are found to be associated with protection against enteric fever.	[17,18]
***HLA*** **Gene Complex Class II and Class III**	Chromosome 6	HLA-DRB1*04:05 and TNF*1 (−308) allele is associated with resistance to enteric fever whereas HLA-DRB1*0301/6/8 and HLA-DQB1*0201-3 allele are associated with susceptibility to enteric fever. TNFA*2 (−308) is associated with the outcome of *Salmonella* typhoidal species infection.	[19,20,21]
**Gene Variants that Do Not Affect the Enteric Fever Outcomes**			
***TLR5***	Chromosome 1	*TLR5* variants do not have a significant effect on the susceptibility or severity of enteric fever.	[22,23]
***NRAMP1***	Chromosome 2	*NRAMP1* polymorphisms are not associated with acquiring enteric fever.	[24]

^a^ The studies were conducted at the endemic regions of the countries/continent (such as Malaysia, Vietnam, Africa, India, Indonesia and Netherlands) for each polymorphism.
